# Left Ventricular Global Longitudinal Strain and Ventricular Repolarization in People with HIV: A Comparative Cross-Sectional Study

**DOI:** 10.3390/medicina62102012

**Published:** 2026-10-18

**Authors:** Elçin Özdemir Tutar, Yunus Emre Özbebek, Zeynep Seyma Turinay Ertop, Semiha Solak Grassie

**Affiliations:** 1 Department of Cardiology, Yenimahalle Training and Research Hospital, 06370 Ankara, Türkiye; 2 Department of Cardiology, Ankara Etlik City Hospital, 06170 Ankara, Türkiye; drozbebek@gmail.com; 3 Department of Cardiology, Ankara Medicana International Hospital, 06510 Ankara, Türkiye; zs_turinay@hotmail.com; 4 Department of Infectious Diseases and Clinical Microbiology, Yenimahalle Training and Research Hospital, 06370 Ankara, Türkiye; semihasolak@yahoo.com

**Keywords:** HIV, global longitudinal strain, speckle-tracking echocardiography, Tp-e interval, ventricular repolarization

## Abstract

*Background and Objectives:* Subclinical myocardial deformation and ventricular repolarization abnormalities may occur in people with HIV (PWH) despite preserved left ventricular ejection fraction (LVEF). We evaluated left ventricular global longitudinal strain (LV GLS) and Tpeak-to-end (Tp-e)-based repolarization indices in PWH and examined their within-person relationship. *Materials and Methods:* This prospective comparative cross-sectional study included 25 PWH and 47 HIV-negative reference participants consecutively recruited from outpatient clinics. All participants were in sinus rhythm and had LVEF ≥50%. Conventional and speckle-tracking echocardiography and 12-lead electrocardiography were performed. Multivariable linear regression assessed associations of HIV status with LV GLS, Tp-e interval, and Tp-e/QTc ratio after adjustment for age, sex, current smoking, and hyperlipidemia. Within-PWH associations were assessed using Spearman correlation with multiplicity adjustment. *Results:* PWH had less negative LV GLS than reference participants (−17.1% vs. −19.0%; *p* < 0.001), longer Tp-e intervals (74 vs. 72 ms; *p* = 0.001), and higher Tp-e/QTc ratios (0.185 vs. 0.173; *p* < 0.001). HIV status remained associated with LV GLS (B = 2.472 percentage points, 95% CI 1.672–3.272), Tp-e (B = 4.347 ms, 95% CI 2.286–6.408), and Tp-e/QTc (B = 0.013, 95% CI 0.009–0.017; all *p* < 0.001). Within PWH, LV GLS was not significantly correlated with Tp-e or Tp-e/QTc. *Conclusions:* PWH with preserved LVEF showed less negative LV GLS and less favorable Tp-e-based repolarization indices than HIV-negative reference participants. These cross-sectional findings do not establish causal, prognostic, or screening implications, and the non-significant within-PWH correlations do not exclude potentially relevant associations. Larger longitudinal studies are warranted.

## 1. Introduction

The widespread use of effective antiretroviral therapy (ART) has substantially improved survival among people with HIV (PWH), shifting the long-term disease burden toward chronic non-AIDS comorbidities, including cardiovascular disease. Beyond conventional cardiovascular risk factors, persistent immune activation, chronic inflammation, endothelial dysfunction, metabolic disturbances, and treatment-related effects may contribute to cardiovascular involvement in this population [1]. Although overt HIV-associated cardiomyopathy has become less prominent in the contemporary ART era, subclinical cardiac abnormalities remain relevant. Echocardiographic studies have demonstrated systolic and diastolic alterations in PWH, including subtle abnormalities despite preserved left ventricular ejection fraction (LVEF) [2,3]. More broadly, subclinical cardiovascular dysfunction may also be reflected by circulating biomarkers such as high-sensitivity cardiac troponin and natriuretic peptides, supporting a multidimensional approach to the detection of early cardiovascular involvement [4].

Speckle-tracking echocardiography enables quantitative assessment of myocardial deformation through left ventricular global longitudinal strain (LV GLS) and may identify subtle systolic dysfunction not apparent on conventional echocardiography. The sensitivity of GLS to subclinical myocardial impairment despite preserved conventional systolic function has also been demonstrated in non-HIV populations, including patients with type 2 diabetes mellitus and individuals with frequent premature ventricular complexes but no structural heart disease [5,6]. Previous studies have reported less favorable LV strain in PWH with preserved LVEF [7,8], although findings have not been uniform across populations, particularly after adjustment for cardiovascular risk factors [9]. Cardiac magnetic resonance studies have also demonstrated myocardial tissue abnormalities in selected PWH, further supporting the presence of subclinical myocardial involvement [10]. Together, these findings suggest that LV myocardial deformation may provide additional information on early cardiac involvement in HIV.

Altered ventricular repolarization may represent another component of this cardiovascular phenotype. The interval from the peak to the end of the T wave (Tp-e) and Tp-e-derived ratios have been used as surface-electrocardiographic indices of ventricular repolarization heterogeneity. Several studies have reported longer Tp-e intervals and higher Tp-e-derived ratios in PWH than in HIV-negative controls [11,12,13,14], while larger cohort data have also linked repolarization variability with HIV status, viraemia, and inflammatory markers [15]. Despite evidence for both myocardial deformation abnormalities and altered ventricular repolarization in PWH, these domains have largely been examined separately. Studies in non-HIV populations suggest potential links between impaired myocardial mechanics and repolarization indices [16,17]. However, in the literature identified for the present study, the within-person association between LV GLS and Tp-e-based measures in PWH had not been quantified.

Accordingly, the present study aimed to evaluate the relationship between LV GLS and ventricular repolarization indices, particularly Tp-e interval and Tp-e/QTc ratio, in PWH, while secondarily comparing myocardial deformation, repolarization, and conventional echocardiographic findings between PWH and HIV-negative reference participants.

## 2. Materials and Methods

### 2.1. Study Design and Population

This prospective comparative cross-sectional study was conducted at a tertiary-care hospital between 15 April and 31 July 2026. During the study period, potentially eligible PWH and HIV-negative reference participants were screened consecutively within their respective recruitment settings. PWH were referred from the Infectious Diseases outpatient clinic for routine cardiac evaluation and were either newly diagnosed (defined as HIV diagnosis within the preceding 3 months) or receiving antiretroviral therapy (ART). The HIV-negative reference group was recruited from individuals attending the cardiology outpatient clinic for routine cardiovascular evaluation who were asymptomatic and had no known structural heart disease or clinically significant cardiac abnormality on screening evaluation. Eligible individuals who provided informed consent were enrolled. All participants were aged ≥18 years, were in sinus rhythm, and had a left ventricular ejection fraction (LVEF) ≥50%.

**Figure 1:**
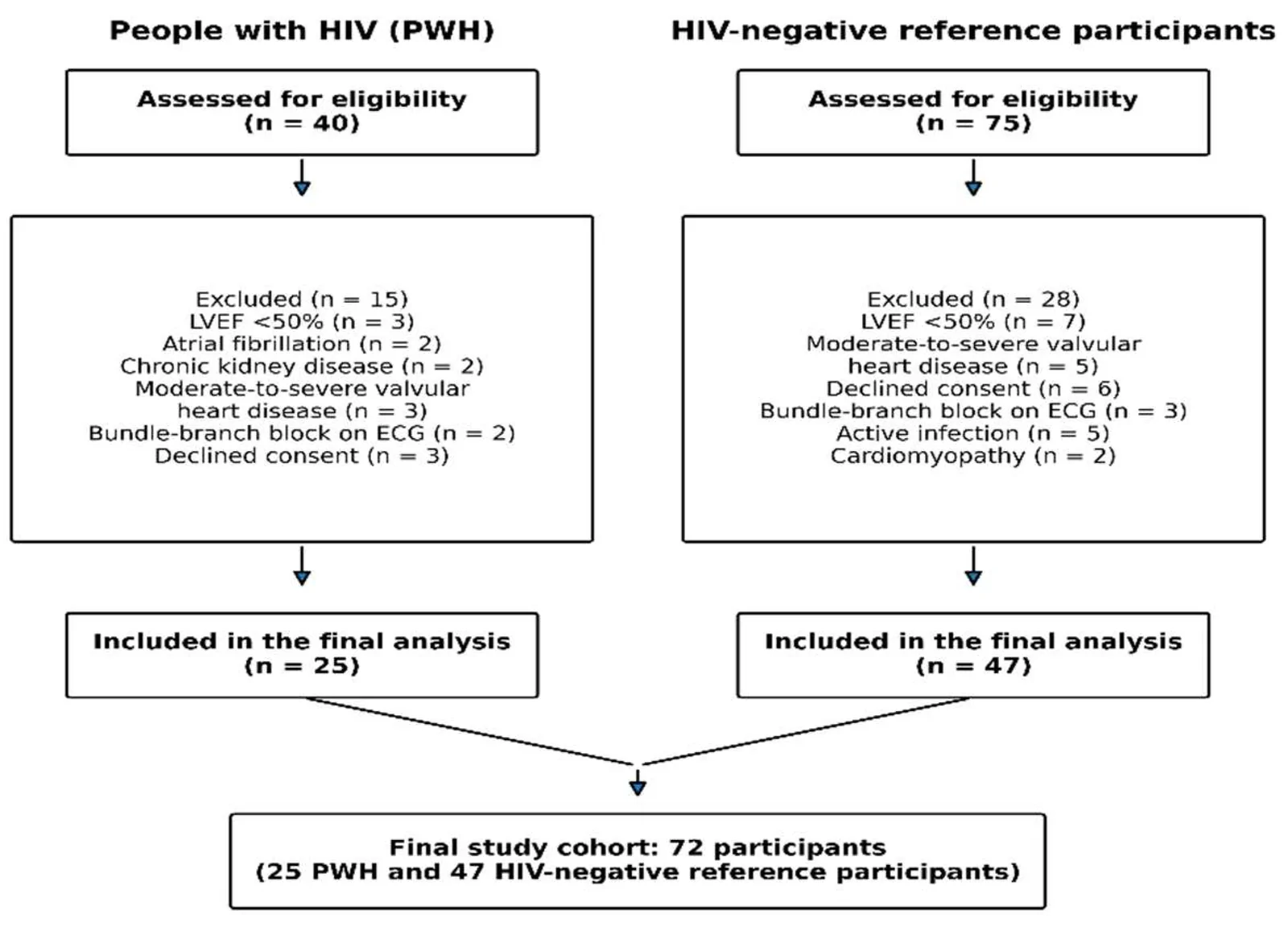
Participant flow from eligibility assessment to final inclusion in the study. ECG, electrocardiogram; HIV, human immunodeficiency virus; LVEF, left ventricular ejection fraction; PWH, people with HIV.

Exclusion criteria for both groups included LVEF <50% on the screening echocardiographic examination performed at study enrollment, moderate-to-severe valvular heart disease, cardiomyopathy, atrial fibrillation or flutter, bundle-branch block or paced rhythm, congenital heart disease, moderate-to-severe pulmonary hypertension, chronic kidney disease with an estimated glomerular filtration rate (eGFR) <30 mL/min/1.73 m^2^, acute systemic infection or febrile illness, previous cardiotoxic chemotherapy, pregnancy, and inadequate echocardiographic image quality. Electrocardiograms unsuitable for reliable interval measurements were excluded from the corresponding analyses. A total of 115 individuals were assessed for eligibility; participant screening, reasons for exclusion, and final inclusion are summarized in Figure 1.

Demographic characteristics, body mass index, smoking status, cardiovascular risk factors, comorbidities, and relevant medical history were recorded prospectively. In PWH, CD4+ T-cell count, viral load status, ART duration, and current ART regimen were additionally recorded. Laboratory and HIV-related data obtained within 30 days of cardiac evaluation were used for analysis. All participants underwent transthoracic echocardiography, LV speckle-tracking analysis, and standard 12-lead electrocardiography on the same day.

### 2.2. Echocardiographic Examination and Speckle-Tracking Analysis

Transthoracic echocardiography was performed using a Philips Affiniti 50 ultrasound system (Philips Healthcare, Andover, MA, USA) in accordance with the joint American Society of Echocardiography/European Association of Cardiovascular Imaging recommendations [18]. Standard chamber dimensions, LVEF, LAVI, LVMI, transmitral Doppler parameters, mitral annular tissue-Doppler velocities, TAPSE, tricuspid annular S′ velocity, and pulmonary artery systolic pressure were obtained using guideline-recommended techniques. LV diastolic function was assessed according to the 2025 ASE recommendations using mitral inflow, mitral annular e′ velocities, average E/e′, LAVI, and pulmonary artery systolic pressure [19]. Grade 1 diastolic dysfunction was defined as impaired relaxation with preserved estimated filling pressures according to the ASE algorithm.

For speckle-tracking analysis, grayscale images from the apical four-chamber, two-chamber, and long-axis views were acquired at 50–70 frames/s and analyzed offline using vendor-independent TomTec software (TOMTEC-ARENA, version 2.51.00; Imaging Systems GmbH, Unterschleissheim, Germany). The target frame rate of 50–70 frames/s was achieved in all studies included in the speckle-tracking analysis, and no study was excluded solely because of inadequate frame rate. LV GLS was derived from the standard 17-segment model across the three apical views and reported as a signed percentage, with more negative values indicating better longitudinal deformation.

### 2.3. Electrocardiographic Assessment

Standard resting 12-lead electrocardiograms were recorded in the supine position after at least 10 min of rest using a Cardiofax M electrocardiograph (ECG-1350K, Nihon Kohden Corporation, Tokyo, Japan) at 25 mm/s and 10 mm/mV. Recordings with excessive artifact, baseline instability, interfering ectopic beats, or inadequate T-wave morphology were excluded from the corresponding measurements.

Heart rate, corrected QT interval (QTc), QRS duration, and Tpeak-to-end (Tp-e) interval were assessed. The QT interval was measured from QRS onset to the end of the T wave, which was defined using the tangent method. QTc was calculated using Bazett’s formula [20]. In addition to Bazett’s correction, QTc was calculated from the QT interval and heart rate using Fridericia’s formula (QTcF = QT/RR^1/3^), with RR calculated as 60/heart rate in seconds, for sensitivity analysis [21].

The Tp-e interval was measured manually on digitally displayed ECGs from the peak to the end of the T wave. Lead V5 was used preferentially, consistent with previous methodological studies, with V6 used when reliable T-wave delineation was not possible in V5 [20]. Measurements were performed using digital magnification and electronic calipers and averaged over three consecutive cycles within the selected lead. T-wave termination was defined by the tangent method. Tp-e/QTc and QTc/QRS were calculated as the ratios of Tp-e to QTc and QTc to QRS duration, respectively; QTc/QRS was considered an exploratory repolarization-to-depolarization index.

### 2.4. Reproducibility Analysis

Intraobserver and interobserver reproducibility of LV GLS and Tp-e measurements was assessed in 20 randomly selected participants. The primary observer repeated measurements after at least 2 weeks, and a second blinded observer independently re-evaluated the same studies. Agreement was assessed using ICCs from a two-way random-effects, absolute-agreement, single-measurement model and Bland–Altman analysis. The primary observer’s measurements were used for the main analyses.

### 2.5. Ethics Approval and Consent to Participate

The study was conducted in accordance with the Declaration of Helsinki and was approved by the Ankara Yenimahalle Training and Research Hospital Clinical Research Ethics Committee (approval no. E-2026-10; 9 April 2026). Written informed consent was obtained from all participants before enrollment.

### 2.6. Sample Size Considerations

No formal a priori sample-size calculation was performed. All consecutively eligible participants recruited during the predefined study period were included. For interpretive purposes, an approximate post hoc power assessment was performed for the within-PWH correlation analyses; with *n* = 25 and a two-sided α of 0.025, the available sample provided approximately 63% power to detect a correlation of |ρ| = 0.50. This assessment was intended to contextualize the precision of the null findings rather than to serve as a formal prospective power calculation.

### 2.7. Statistical Analysis

Statistical analyses were performed using IBM SPSS Statistics version 29.0 (IBM Corp., Armonk, NY, USA). Continuous variables are presented as mean ± standard deviation or median [interquartile range], as appropriate, and categorical variables as *n* (%). Between-group comparisons were performed using the independent-samples t test, Welch’s t test, Mann–Whitney U test, Pearson’s chi-square test, or Fisher’s exact test, as appropriate.

The principal study measures were LV GLS, Tp-e interval, and Tp-e/QTc ratio. Multivariable linear regression models were used to assess their associations with HIV status after adjustment for age, sex, current smoking, and hyperlipidemia. Results are reported as unstandardized coefficients (B), 95% confidence intervals (CIs), standardized β coefficients, and *p* values.

Several sensitivity analyses were performed. First, the primary models were repeated after exclusion of the three participants with diabetes mellitus, which was not included in the primary models because of its sparse and completely unbalanced distribution. Second, Tp-e was additionally modeled with QTc entered as a covariate. Third, exploratory expanded models additionally adjusted for LVMI, BMI, and heart rate. Effect modification by HIV status was assessed in the full cohort using interaction terms between mean-centered LV GLS and HIV status in models for Tp-e and Tp-e/QTc.

Within PWH, associations of LV GLS with Tp-e and Tp-e/QTc were assessed using Spearman’s rank correlation. Because these two correlations addressed the same hypothesis, a Bonferroni-adjusted significance threshold of *p* < 0.025 was applied; 95% CIs were estimated using BCa bootstrap resampling with 10,000 participant-level resamples. Exploratory associations of CD4+ T-cell count, ART duration, and CRP with LV GLS, Tp-e, and Tp-e/QTc were also examined using Spearman’s correlation, with Benjamini–Hochberg false-discovery-rate correction across the nine tests. Viral load status and ART regimens were summarized descriptively because of the small subgroup sizes.

Multicollinearity and regression diagnostics were assessed using variance inflation factors and standard residual/influence diagnostics. No data imputation was performed. All tests were two-sided, with *p* < 0.05 considered statistically significant unless otherwise specified.

## 3. Results

### 3.1. Study Population

After exclusions, 25 PWH and 47 HIV-negative reference participants were included in the final analysis (Figure 1). Age, sex, body mass index, current smoking, and systolic and diastolic blood pressure did not differ significantly between groups. Hyperlipidemia (40.0% vs. 17.0%; *p* = 0.032) and diabetes mellitus (12.0% vs. 0%; *p* = 0.039) were more frequent among PWH, whereas hypertension and coronary artery disease did not differ. Antihypertensive and statin use were comparable between groups; antidiabetic medication was used only among PWH (Table 1).

### 3.2. HIV-Related Clinical Characteristics

Among PWH, the median CD4+ T-cell count was 562 [399–772] cells/µL and the median duration of antiretroviral therapy (ART) was 24 [3–33] months. Viral load was undetectable in 20 participants (80.0%) and detectable in 5 (20.0%); all participants with detectable viraemia were newly diagnosed and had recently initiated ART. Detailed ART regimens and HIV-related laboratory characteristics are presented in Table 2.

### 3.3. Echocardiographic Findings

LVEF was similar between PWH and HIV-negative reference participants (65 [59–65]% vs. 65 [62–65]%; *p* = 0.085). LVMI was higher and mitral E-wave velocity was lower in PWH (*p* = 0.036 and *p* = 0.014, respectively), while the remaining conventional echocardiographic measures showed no significant between-group differences (Table 3).

**Figure 2:**
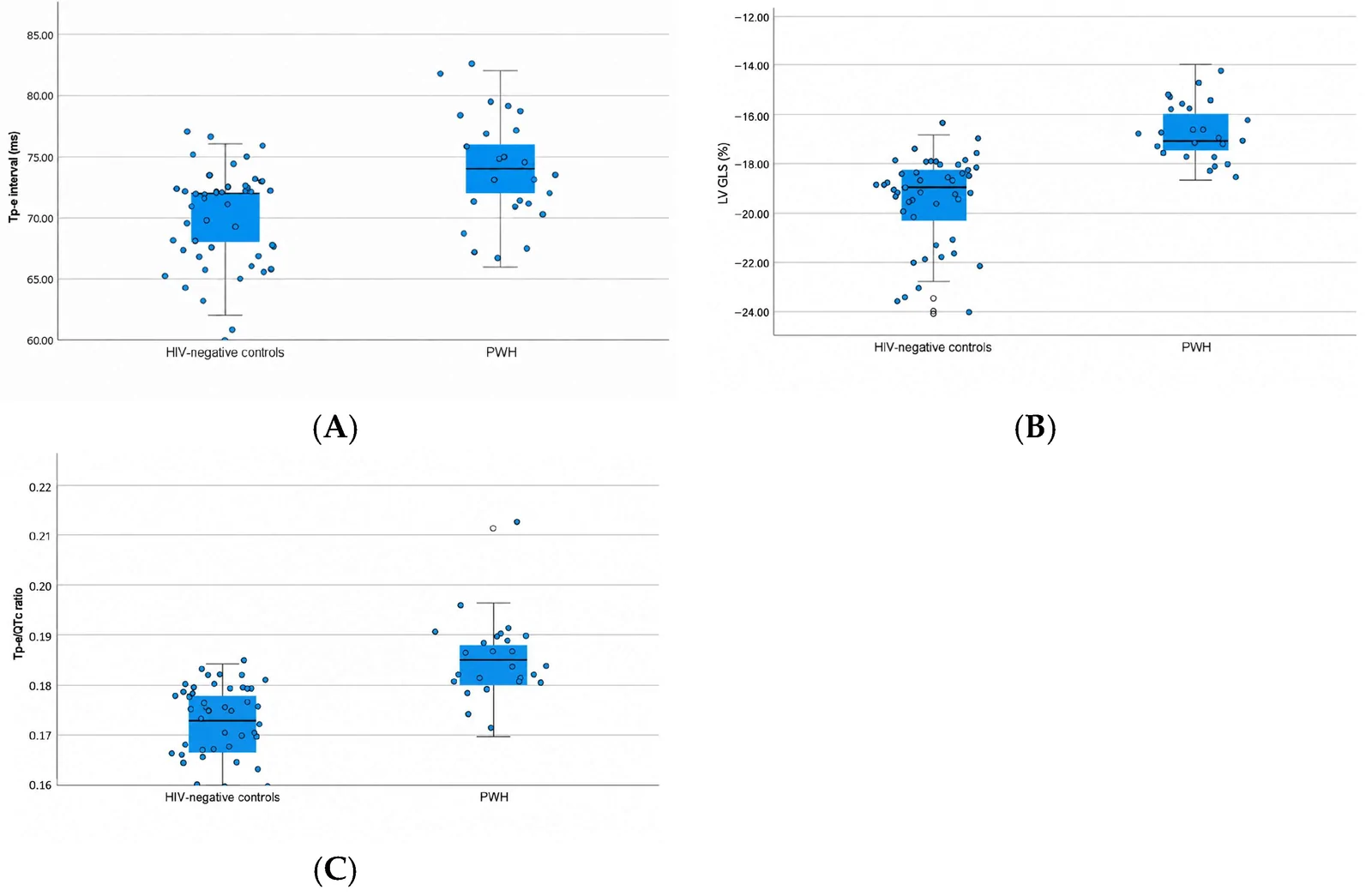
Distribution of principal myocardial deformation and ventricular repolarization measures according to HIV status. Individual data points are shown with box plots representing the median and interquartile range: (**A**) Tp-e interval, (**B**) LV GLS, and (**C**) Tp-e/QTc ratio. Abbreviations: LV GLS, left ventricular global longitudinal strain; PWH, people with HIV; Tp-e, Tpeak-to-end interval.

LV GLS was less negative in PWH than in HIV-negative reference participants (−17.1 [−17.5 to −16.0]% vs. −19.0 [−20.5 to −18.3]%; *p* < 0.001; Figure 2A). Grade 1 diastolic dysfunction was identified in 2 PWH (8.0%) and 1 reference participant (2.1%; *p* = 0.275), and no participant met criteria for Grade 2 or Grade 3 diastolic dysfunction.

### 3.4. Electrocardiographic Findings

PWH had a longer Tp-e interval (74 [71–77] vs. 72 [68–72] ms; *p* = 0.001) and a higher Tp-e/QTc ratio (0.185 [0.180–0.188] vs. 0.173 [0.166–0.178]; *p* < 0.001) than HIV-negative reference participants (Figure 2B,C). Heart rate, QTc interval, and QRS duration did not differ between groups, whereas the QTc/QRS ratio was lower in PWH (4.39 ± 0.51 vs. 4.69 ± 0.56; *p* = 0.029) (Table 4).

### 3.5. Multivariable Analyses

After adjustment for age, sex, current smoking, and hyperlipidemia, HIV status was associated with less negative LV GLS (B = 2.472 percentage points, 95% CI 1.672–3.272; *p* < 0.001), longer Tp-e interval (B = 4.347 ms, 95% CI 2.286–6.408; *p* < 0.001), and higher Tp-e/QTc ratio (B = 0.013, 95% CI 0.009–0.017; *p* < 0.001) (Table 5).

### 3.6. Sensitivity and Exploratory Analyses

Model diagnostics are summarized in Supplementary Table S1 and Supplementary Figure S1. Although maximum absolute standardized residuals exceeded 3 in the LV GLS and Tp-e/QTc models, maximum Cook’s distances ranged from 0.101 to 0.147.

Sensitivity analyses supported the principal findings. After exclusion of the three participants with diabetes mellitus, HIV status remained associated with LV GLS (B = 2.433 percentage points, 95% CI 1.591–3.276; *p* < 0.001), Tp-e interval (B = 4.288 ms, 95% CI 2.144–6.433; *p* < 0.001), and Tp-e/QTc ratio (B = 0.013, 95% CI 0.009–0.018; *p* < 0.001) (Supplementary Table S2).

With Fridericia correction, QTc remained similar between groups, the Tp-e/QTc ratio remained higher in PWH, and the between-group difference in QTc/QRS was no longer significant (Supplementary Table S3). In a separate model including QTc as a covariate, HIV status remained associated with Tp-e interval (B = 4.93 ms, 95% CI 3.29–6.57; *p* < 0.001).

In exploratory models additionally adjusted for LVMI, BMI, and heart rate, HIV status remained associated with LV GLS (B = 2.69 percentage points, 95% CI 1.89–3.49; *p* < 0.001), Tp-e interval (B = 3.63 ms, 95% CI 1.58–5.68; *p* = 0.001), and Tp-e/QTc ratio (B = 0.0115, 95% CI 0.0075–0.0155; *p* < 0.001); the maximum VIF was 1.35.

The GLS × HIV interaction was not significant for Tp-e interval (B = 0.116, 95% CI −1.468 to 1.700; *p* = 0.884) or Tp-e/QTc ratio (B = −0.0011, 95% CI −0.0043 to 0.0022; *p* = 0.522).

### 3.7. Within-PWH Relationship Between LV Myocardial Deformation and Ventricular Repolarization

Among PWH (*n* = 25), LV GLS was not significantly correlated with Tp-e interval (Spearman’s ρ = −0.056, 95% BCa bootstrap CI −0.451 to 0.368; *p* = 0.790) or Tp-e/QTc ratio (ρ = −0.231, 95% BCa bootstrap CI −0.547 to 0.132; *p* = 0.267). Neither correlation met the Bonferroni-adjusted significance threshold of *p* < 0.025.

### 3.8. Exploratory HIV-Related Correlates

None of the nine exploratory correlations of CD4+ T-cell count, ART duration, or CRP with LV GLS, Tp-e interval, or Tp-e/QTc ratio reached statistical significance before or after Benjamini–Hochberg correction (all unadjusted *p* ≥ 0.179; all FDR-adjusted *p* ≥ 0.877), and all 95% bootstrap confidence intervals included zero. Complete estimates and bootstrap confidence intervals are provided in Supplementary Table S4.

Descriptive values for the newly diagnosed/viremic subgroup (*n* = 5) and the virologically suppressed subgroup (*n* = 20) are provided in Supplementary Table S5.

### 3.9. Reproducibility

Reproducibility analyses demonstrated high agreement for both LV GLS and Tp-e measurements. The mean intraobserver and interobserver biases were 0.20 and 0.10 ms for Tp-e and 0.07 and 0.00 percentage points for LV GLS, respectively. The corresponding 95% limits of agreement were narrow, and ICC values ranged from 0.990 to 0.999. Detailed reproducibility estimates are provided in Supplementary Table S6.

## 4. Discussion

The principal findings of this prospective comparative cross-sectional study were that PWH with preserved LVEF had less negative LV GLS, longer Tp-e intervals, and higher Tp-e/QTc ratios than HIV-negative reference participants. These associations remained evident after adjustment for age, sex, current smoking, and hyperlipidemia and were broadly consistent across the reported sensitivity analyses. In contrast, no statistically significant within-PWH association was identified between LV GLS and either Tp-e interval or Tp-e/QTc ratio. Current CD4+ T-cell count, ART duration, and CRP were likewise not significantly associated with the principal cardiac measures. These findings describe cross-sectional associations and should be considered hypothesis-generating rather than causal or prognostic evidence.

Previous HIV strain studies have yielded heterogeneous findings. Our observation of less negative LV GLS despite preserved LVEF is consistent with speckle-tracking studies reporting impaired longitudinal deformation in PWH [7,8]. Athanasiadi et al. reported a greater prevalence of GLS-defined subclinical LV systolic dysfunction among ART-treated PWH than among matched controls, with adiposity and carotid atherosclerosis associated with impaired GLS [8]. Liao et al. also demonstrated differences in myocardial deformation among PWH, with less favorable LV GLS in ART-naive than ART-experienced participants despite preserved conventional systolic function [7]. Conversely, Hoy et al. did not detect a statistically significant adjusted GLS difference between asymptomatic PWH receiving long-term ART and HIV-negative participants [9]. Differences in cohort composition, virological suppression, treatment history, cardiovascular risk profiles, and imaging methodology complicate direct comparison across studies. Cardiac magnetic resonance studies demonstrating myocardial oedema and fibrosis in selected PWH provide additional biological context, although tissue characterization was not performed in the present study [10].

The approximately 1.9 percentage-point between-group difference in LV GLS represents a group-level shift whose clinical importance cannot be determined from the present data. General ASE/EACVI consensus categories consider LV GLS values more negative than −18% generally normal, values between −16% and −18% borderline, and values less negative than −16% abnormal [22]. The group medians in the present study therefore lie within the borderline and generally normal ranges, respectively, but these categories do not establish individual myocardial dysfunction, an HIV-specific diagnostic threshold, or the clinical significance of the between-group difference. GLS is also influenced by loading conditions and technical factors. Although systolic and diastolic blood pressures were similar between groups, residual differences in loading conditions cannot be excluded, and comparison with other cohorts should take acquisition and analysis methodology into account [22].

The repolarization findings are also consistent with previous HIV studies reporting longer Tp-e intervals and higher Tp-e-derived ratios [11,12,13,14]. Ünal et al. and Unal Evren et al. reported longer Tp-e and higher Tp-e-derived ratios in PWH, with associations also described between repolarization indices and immunological or inflammatory measures [11,13]. Çetin et al. similarly observed altered Tp-e-based indices in ART-treated participants, including virologically suppressed individuals [12,14]. In contrast to these Tp-e-based studies, Heravi et al. evaluated beat-to-beat QT variability in the Multicenter AIDS Cohort Study and demonstrated a broader repolarization phenotype associated with HIV status, viraemia, and inflammatory markers [15]. These findings support the presence of electrical repolarization abnormalities in selected PWH populations but do not establish prognostic significance for Tp-e in the present cohort.

The absolute Tp-e difference observed here was modest, and Tp-e-based measures should be regarded as surface-ECG surrogate indices rather than direct measures of arrhythmic risk. Nevertheless, HIV status remained associated with longer Tp-e when QTc was entered directly as a covariate, indicating that the observed Tp-e difference was not solely dependent on use of the Tp-e/QTc ratio. The Tp-e/QTc difference also persisted after Fridericia correction. In contrast, the between-group difference in QTc/QRS was attenuated and was no longer statistically significant when Fridericia correction was applied, suggesting that this exploratory comparison was less robust to the QT-correction approach. These sensitivity analyses strengthen the consistency of the observed repolarization findings but do not establish a specific electrophysiological mechanism.

The present study also examined LV GLS and Tp-e-based indices together within the same PWH cohort. Studies in non-HIV populations have reported associations between myocardial deformation and ECG-derived repolarization measures [16,17], providing a rationale for evaluating these domains jointly. However, neither the GLS–Tp-e nor GLS–Tp-e/QTc correlation reached the Bonferroni-adjusted significance threshold in PWH. Similarly, the GLS × HIV interaction terms were not statistically significant for either repolarization measure in the full cohort. These analyses do not establish that the relationships are equivalent across HIV-status groups or that no underlying association exists. The within-PWH sample included only 25 participants, and the confidence intervals were sufficiently wide to leave potentially relevant associations unresolved.

Cardiovascular risk-factor imbalance remains an important potential source of confounding. Hyperlipidemia was more frequent among PWH and was included in the primary multivariable models. Diabetes mellitus occurred exclusively among PWH and could not be reliably included in the primary models because only three participants had diabetes. Diabetes is itself associated with subclinical myocardial dysfunction, including abnormalities in LV GLS [6]. However, exclusion of these three participants yielded very similar HIV-status coefficients for LV GLS, Tp-e interval, and Tp-e/QTc ratio. The principal associations also remained evident in exploratory models additionally adjusted for LVMI, BMI, and heart rate. These findings indicate stability of the estimates across the reported sensitivity analyses, but they do not eliminate residual confounding from measured or unmeasured factors.

The exploratory HIV-related analyses also require cautious interpretation. Current CD4+ T-cell count, ART duration, and CRP were not significantly associated with LV GLS, Tp-e interval, or Tp-e/QTc ratio after false-discovery-rate correction, and all corresponding 95% bootstrap confidence intervals included zero. This differs from some previous reports describing associations of Tp-e-based measures with nadir CD4 count or high-sensitivity CRP [13,14]. However, nadir CD4 was unavailable in the present study, routine rather than high-sensitivity CRP was measured, and most participants were virologically suppressed. In addition, recorded ART duration may not reflect cumulative lifetime exposure. These findings therefore should not be interpreted as evidence that immune status, inflammatory burden, or treatment exposure are unrelated to myocardial or electrical phenotype.

The PWH cohort was also heterogeneous, including a small newly diagnosed/viremic subgroup and a larger group receiving ART with virological suppression. These groups may differ in inflammatory burden, duration of HIV exposure, and treatment-related effects. The small size of the newly diagnosed/viremic subgroup precluded reliable inferential comparisons and prevents conclusions regarding subgroup equivalence. Pooling these participants may have obscured subgroup-specific associations, but the present sample cannot determine whether such differences exist or their direction.

Strengths of the study include prospective enrollment, standardized three-view 17-segment vendor-independent GLS analysis, standardized Tp-e measurement with digital magnification and three-cycle averaging, blinded assessment, reproducibility evaluation, and complementary sensitivity analyses. Several limitations should also be acknowledged. This was a single-center study with only 25 PWH, limiting precision and generalizability. Although participants were enrolled prospectively, the comparative analyses were cross-sectional and cannot establish whether HIV exposure preceded the observed cardiac differences or whether myocardial deformation and repolarization changes are causally related. The HIV-negative reference group was recruited from a cardiology outpatient population rather than from the general community, which may have introduced selection bias of uncertain direction. Tp-e measurements were obtained preferentially from V5, with V6 used when V5 was unsuitable, and repolarization variation across other precordial leads was therefore not assessed. Nadir CD4 count and cumulative lifetime ART exposure were unavailable, detectable viraemia was uncommon, and routine rather than high-sensitivity CRP was measured. The sample was insufficient for meaningful comparisons among individual ART strategies, and cardiac magnetic resonance and specific biomarkers of myocardial inflammation or fibrosis were unavailable. Finally, although multiplicity correction was applied to the within-PWH and exploratory HIV-related correlation families, no global correction was applied across all secondary and sensitivity analyses; these findings should therefore be considered supportive or exploratory.

The present data do not support routine use of LV GLS or Tp-e-based indices for cardiovascular screening or risk stratification in PWH, because diagnostic performance and clinical outcomes were not evaluated. No ventricular arrhythmic events, heart-failure outcomes, or prognostic thresholds were assessed. Larger, adequately powered longitudinal studies using standardized myocardial deformation and repolarization measurements and adjudicated cardiovascular outcomes are needed to determine the clinical relevance of these hypothesis-generating associations.

## 5. Conclusions

In this prospective comparative cross-sectional study, PWH with preserved LVEF had less negative LV GLS, longer Tp-e intervals, and higher Tp-e/QTc ratios than HIV-negative reference participants. These findings represent group-level associations and do not establish causal, prognostic, or screening implications. No statistically significant within-PWH association was detected between LV GLS and Tp-e-based repolarization measures; however, the small sample size and wide confidence intervals preclude exclusion of potentially relevant underlying relationships. These hypothesis-generating findings warrant larger, adequately powered longitudinal studies with clinical cardiovascular outcomes to determine their clinical significance.

## Figures and Tables

**Table 1:** Baseline demographic and clinical characteristics of the study population.

Variable	Controls (*n* = 47)	People Living with HIV (*n* = 25)	*p* Value
Age, years	43.43 ± 9.48	46.08 ± 14.04	0.402
Male sex, *n* (%)	27 (57.4)	19(76.0)	0.119
BMI, kg/m^2^	24.7 [23.3–27.8]	25.7 [22.9–28.7]	0.972
Systolic blood pressure, mmHg	108.28 ± 11.35	111.68 ± 7.95	0.187
Diastolic blood pressure, mmHg	68 [65–76]	71 [65–77]	0.299
Hypertension, *n* (%)	7 (14.9)	3 (12.0)	1.000
Diabetes mellitus, *n* (%)	0 (0.0)	3 (12.0)	0.039
Hyperlipidemia, *n* (%)	8 (17.0)	10 (40.0)	0.032
Current smoking, *n* (%)	19 (40.4)	15 (60.0)	0.113
Coronary artery disease, *n* (%)	0 (0.0)	1 (4.0)	0.347
Antihypertensive medication, *n* (%)	7 (14.9)	3 (12.0)	1.000
Antidiabetic medication, *n* (%)	0 (0.0)	3 (12.0)	0.039
Statin therapy, *n* (%)	5 (10.6)	4 (16.0)	0.710

Data are presented as mean ± standard deviation, median [interquartile range], or *n* (%), as appropriate. Abbreviation: BMI, body mass index.

**Table 2:** Laboratory and treatment characteristics of People living with HIV.

Variable	People Living with HIV (*n* = 25)
CD4+ T-cell count, cells/µL	562 [399–772]
Detectable viral load, *n* (%)	5 (20.0)
Undetectable viral load, *n* (%)	20 (80.0)
ART duration, months	24 [3–33]
LDL-C, mg/dL	115.4 ± 34.8
Triglycerides, mg/dL	142.2 ± 61.6
HDL-C, mg/dL	46.0 ± 9.9
WBC count, ×10^9^/L	6.52 ± 1.79
Lymphocyte count, ×10^9^/L	2.21 ± 0.67
Creatinine, mg/dL	0.98 ± 0.16
eGFR, mL/min/1.73 m^2^	92.8 ± 17.4
HbA1c, %	5.62 ± 0.53
CRP, mg/L	2.5 [1.5–8.4]
TSH, mIU/L	2.06 ± 0.82
INSTI + two NRTIs, *n* (%)	20 (80.0)
Boosted INSTI + two NRTIs, *n* (%)	3 (12.0)
INSTI + one NRTI dual therapy, *n* (%)	2 (8.0)

Data are presented as mean ± standard deviation, median [interquartile range], or *n* (%), as appropriate. The detectable viral load subgroup (*n* = 5) consisted entirely of newly diagnosed participants who had recently initiated ART. The individual ART regimens were Biktarvy in 10 patients (40.0%), Truvada plus Tivicay in 8 (32.0%), Genvoya in 3 (12.0%), Triumeq in 2 (8.0%), Tivicay plus Epivir in 1 (4.0%), and Dovipsa in 1 (4.0%). Abbreviations: ART, antiretroviral therapy; CRP, C-reactive protein; eGFR, estimated glomerular filtration rate; HbA1c, glycated hemoglobin; HDL-C, high-density lipoprotein cholesterol; INSTI, integrase strand transfer inhibitor; LDL-C, low-density lipoprotein cholesterol; NRTI, nucleoside/nucleotide reverse transcriptase inhibitor; TSH, thyroid-stimulating hormone; WBC, white blood cell.

**Table 3:** Echocardiographic characteristics of the study groups.

Variable	Controls (*n* = 47)	People Living with HIV (*n* = 25)	*p* Value
LVEF, %	65 [62–65]	65 [59–65]	0.085
LA diameter, cm	3.16 ± 0.47	3.17 ± 0.50	0.917
LAVI, mL/m^2^	14.5 [12.3–16.9]	15.6 [12.6–18.3]	0.241
LVMI, g/m^2^	71.5 [63.5–89.0]	79.0 [69.6–96.1]	0.036
Interventricular septal thickness, cm	0.9 [0.9–1.1]	1.0 [0.9–1.0]	0.458
Mitral E-wave velocity, m/s	0.70 [0.50–0.90]	0.60 [0.50–0.75]	0.014
Mitral A-wave velocity, m/s	0.70 [0.60–0.80]	0.60 [0.50–0.70]	0.099
Septal e′ velocity, cm/s	11 [10–12]	10 [8–12]	0.138
Lateral e′ velocity, cm/s	13 [11–15]	12 [9–14]	0.163
Average E/e′ ratio	6.11 ± 1.21	5.69 ± 1.49	0.202
LV GLS, %	−19.0 [−20.5 to −18.3]	−17.1 [−17.5 to −16.0]	<0.001
TAPSE, cm	2.0 [1.85–2.20]	2.0 [1.80–2.20]	0.820
PASP, mmHg	19.60 ± 5.08	20.56 ± 5.95	0.473
RV tissue Doppler S′ velocity, cm/s	12 [11–13]	12 [12,13]	0.730
Grade 1 diastolic dysfunction, *n* (%)	1 [2.1]	2 [8]	0.275
Mitral E/A ratio	1.25 [0.67–1.50]	0.75 [0.67–1.40]	0.339
E-wave deceleration time, ms	189 [176–217.5]	210 [185–225]	0.097

Data are presented as mean ± standard deviation, median [interquartile range], or *n* (%), as appropriate. Abbreviations: E/e′, ratio of early transmitral flow velocity to early diastolic mitral annular velocity; GLS, global longitudinal strain; LA, left atrial; LAVI, left atrial volume index; LV, left ventricular; LVEF, left ventricular ejection fraction; LVMI, left ventricular mass index; RV, right ventricular; PASP, pulmonary artery systolic pressure; TAPSE, tricuspid annular plane systolic excursion.

**Table 4:** Electrocardiographic characteristics of the study groups.

Variable	Controls (*n* = 47)	People Living with HIV (*n* = 25)	*p* Value
Heart rate, beats/min	80.0 ± 10.1	76.8 ± 12.0	0.231
QTc interval, ms	408.3 ± 20.4	399.5 ± 22.0	0.096
Tp-e interval, ms	72 [68–72]	74 [71–77]	0.001
Tp-e/QTc ratio	0.173 [0.166–0.178]	0.185 [0.180–0.188]	<0.001
QRS duration, ms	88 [80–94]	89.95 [82.98–102.06]	0.220
QTc/QRS ratio	4.69 ± 0.56	4.39 ± 0.51	0.029

Data are presented as mean ± standard deviation or median [interquartile range], as appropriate. Abbreviations: QTc, heart rate-corrected QT interval; Tp-e, interval from the peak to the end of the T wave.

**Table 5:** Multivariable analyses of LV GLS, ventricular repolarization indices.

**(a) Multivariable linear regression for LV GLS**			
**Predictor**	**B (95% CI) percentage points**	**Standardized β**	***p*** **value**
HIV status	2.472 (1.672 to 3.272)	0.567	<0.001
Age, years	0.025 (−0.011 to 0.062)	0.136	0.168
Sex	−0.414 (−1.247 to 0.419)	−0.096	0.324
Current smoking	0.196 (−0.613 to 1.005)	0.047	0.629
Hyperlipidemia	0.656 (−0.308 to 1.619)	0.137	0.179
**(b) Multivariable linear regression for Tp-e interval**			
**Predictor**	**B (95% CI), ms**	**Standardized β**	***p*** **value**
HIV status	4.347 (2.286 to 6.408)	0.478	<0.001
Age, years	0.060 (−0.034 to 0.153)	0.154	0.206
Sex	0.897 (−1.248 to 3.042)	0.099	0.407
Current smoking	−0.495 (−2.579 to 1.589)	−0.057	0.637
Hyperlipidemia	−2.018 (−4.500 to 0.464)	−0.202	0.109
**(c) Multivariable linear regression for Tp-e/QTc ratio**			
**Predictor**	**B (95% CI)**	**Standardized β**	***p*** **value**
HIV status	0.013 (0.009 to 0.017)	0.604	<0.001
Age, years	0.00003 (−0.00016 to 0.00023)	0.035	0.743
Sex	−0.004 (−0.008 to 0.001)	−0.167	0.114
Current smoking	−0.001 (−0.006 to 0.003)	−0.066	0.533
Hyperlipidemia	−0.001 (−0.006 to 0.004)	−0.032	0.771

Model performance: (a) LV GLS model: R^2^ = 0.492; adjusted R^2^ = 0.454; F(5, 66) = 12.800; *p* < 0.001. (b) Tp-e interval model: R^2^ = 0.228; adjusted R^2^ = 0.169; F(5, 66) = 3.893; *p* = 0.004. (c) Tp-e/QTc ratio model: R^2^ = 0.409; adjusted R^2^ = 0.364; F(5, 66) = 9.124; *p* < 0.001. Footnotes: No relevant multicollinearity was identified in the linear regression models; detailed diagnostic statistics are provided in Supplementary Table S1. Abbreviations: B, unstandardized regression coefficient; CI, confidence interval; GLS, global longitudinal strain; LV, left ventricular; QTc, heart rate-corrected QT interval; Tp-e, interval from the peak to the end of the T wave.

## Data Availability

The data presented in this study are available from the corresponding author upon reasonable request. The data are not publicly available due to privacy and ethical considerations.

## Supplementary Materials

The following supporting information can be downloaded at https://www.mdpi.com/article/10.3390/medicina62102012/s1. Supplementary Table S1: Diagnostic statistics for the multivariable linear regression models; Supplementary Table S2: Sensitivity analysis after exclusion of participants with diabetes mellitus; Supplementary Table S3: Electrocardiographic characteristics using Fridericia-corrected QT interval; Supplementary Table S4: Exploratory correlations of HIV-related clinical characteristics and C-reactive protein with myocardial deformation and ventricular repolarization measures among people with HIV; Supplementary Table S5: Descriptive comparison of cardiac measures according to virological status among people with HIV; Supplementary Table S6: Intraobserver and interobserver reproducibility of LV GLS and Tp-e measurements; Supplementary Figure S1: Regression diagnostic plots for the principal multivariable linear regression models, including normal P-P plots of standardized residuals and plots of standardized residuals against standardized predicted values for the LV GLS (**A**,**B**), Tp-e interval (**C**,**D**), and Tp-e/QTc ratio (**E**,**F**) models.
