# Supplementary material for: Left Ventricular Global Longitudinal Strain and Ventricular Repolarization in People with HIV: A Comparative Cross-Sectional Study
Source: Medicina (Kaunas). 2026 Oct 18;62(10):2012. doi: 10.3390/medicina62102012 (PMC13706740; doi:10.3390/medicina62102012)
Supplement: Supplementary file 1 [file medicina-62-02012-s001.zip › medicina-4602732-supplementary.pdf]

Supplementary Tables

Supplementary Table S1. Diagnostic statistics for the multivariable linear regression models

Outcome	VIF range	Tolerance range	Maximum |standardized residual|	Maximum |studentized residual|	Maximum leverage	Maximum Cook's distance
LV GLS	1.099–1.319	0.758–0.910	3.10	3.19	0.184	0.101
Tp-e interval	1.099–1.319	0.758–0.910	2.24	2.36	0.184	0.105
Tp-e/QTc ratio	1.099–1.319	0.758–0.910	3.17	3.30	0.184	0.147

All models included HIV status, age, sex, current smoking, and hyperlipidemia as predictors (n = 72). VIF values ranged from 1.099 to 1.319, and tolerance values ranged from 0.758 to 0.910, indicating no evidence of problematic multicollinearity. These values were identical across models because the same predictors and participants were used. Studentized residuals were internally studentized, and leverage represents the diagonal elements of the hat matrix. Maximum absolute standardized residuals exceeded 3 in the LV GLS and Tp-e/QTc models. Maximum Cook's distances ranged from 0.101 to 0.147; these values summarize potential influence and do not, by themselves, establish that all model assumptions were satisfied.

Abbreviations: VIF, variance inflation factor; GLS, global longitudinal strain; LV, left ventricular; QTc, heart rate-corrected QT interval; Tp-e, interval from the peak to the end of the T wave.

Supplementary Table S2. Sensitivity analysis after exclusion of participants with diabetes mellitus

Outcome	Full cohort (n = 72): adjusted B (95% CI)	p value	Participants without diabetes (n = 69): adjusted B (95% CI)	p value
LV GLS, percentage points	2.472 (1.672 to 3.272)	<0.001	2.433 (1.591 to 3.276)	<0.001
Tp-e interval, ms	4.347 (2.286 to 6.408)	<0.001	4.288 (2.144 to 6.433)	<0.001
Tp-e/QTc ratio	0.01289 (0.00866 to 0.01712)	<0.001	0.01315 (0.00868 to 0.01762)	<0.001

Values represent unstandardized regression coefficients (B) for HIV status from separate multivariable linear regression models for each outcome. All models were adjusted for age, sex, current smoking, and hyperlipidemia. HIV status was coded as 1 for people with HIV and 0 for HIV-negative controls. The full cohort included 25 people with HIV and 47 controls. The post-hoc sensitivity analysis excluded the three participants with diabetes mellitus, all of whom were in the HIV group, leaving 22 people with HIV and 47 controls. There were no missing data for the variables included in these models. LV GLS was analyzed using signed negative values; a positive B therefore indicates less negative LV GLS in people with HIV. Positive coefficients for Tp-e and Tp-e/QTc indicate longer intervals and higher ratios, respectively. Reported p values are two-sided and unadjusted for multiplicity.

Abbreviations: B, unstandardized regression coefficient; CI, confidence interval; GLS, global longitudinal strain; HIV, human immunodeficiency virus; LV, left ventricular; QTc, heart rate-corrected QT interval; Tp-e, interval from the peak to the end of the T wave.

Supplementary Table S3. Electrocardiographic characteristics using Fridericia-corrected QT interval

**Variable**	**Controls (n = 47)**	**People living with HIV (n = 25)**	**p value**
Heart rate, beats/min	80.0 ± 10.1	76.8 ± 12.0	0.231
QTc interval, Fridericia, ms	389.7 ± 19.9	384.2 ± 22.2	0.197
Tp-e interval, ms	72 [68–72]	74 [71–77]	0.001
Tp-e/QTc ratio, Fridericia	0.1798 ± 0.0084	0.1922 ± 0.0061	<0.001
QRS duration, ms	88 [80–94]	89.95 [82.98–102.06]	0.220
QTc/QRS ratio, Fridericia	4.47 ± 0.53	4.22 ± 0.51	0.052

Data are presented as mean ± standard deviation or median [interquartile range], as appropriate. QTc was recalculated using Fridericia's formula as a sensitivity analysis
Abbreviations: QTc, heart rate-corrected QT interval; Tp-e, interval from the peak to the end of the T wave

Supplementary Table S4. Exploratory correlations of HIV-related clinical characteristics and C-reactive protein with myocardial deformation and ventricular repolarization measures among people with HIV.

Variable	Cardiac measure	n	Spearman's ρ	95% bootstrap CI	Unadjusted p value	FDR-adjusted p value
CD4+ T-cell count	LV GLS	25	−0.193	−0.555 to 0.276	0.356	0.877
CD4+ T-cell count	Tp-e interval	25	−0.015	−0.370 to 0.377	0.944	0.944
CD4+ T-cell count	Tp-e/QTc ratio	25	0.079	−0.312 to 0.474	0.707	0.877
ART duration	LV GLS	25	0.150	−0.282 to 0.546	0.475	0.877
ART duration	Tp-e interval	25	0.059	−0.376 to 0.475	0.779	0.877
ART duration	Tp-e/QTc ratio	25	0.277	−0.082 to 0.585	0.179	0.877
CRP	LV GLS	25	−0.077	−0.437 to 0.336	0.714	0.877
CRP	Tp-e interval	25	0.073	−0.291 to 0.472	0.727	0.877
CRP	Tp-e/QTc ratio	25	0.086	−0.346 to 0.497	0.684	0.877

Associations were evaluated using two-sided Spearman's rank correlation tests. LV GLS was analyzed using signed negative values, with less negative values indicating worse longitudinal myocardial deformation. QTc was calculated using Bazett's formula for these analyses. Confidence intervals were estimated using the bias-corrected and accelerated (BCa) bootstrap method with 10,000 participant-level resamples, preserving paired observations. The Benjamini–Hochberg procedure was applied across all nine correlations as a single exploratory family to control the false discovery rate at 5%. Confidence intervals were not adjusted for multiplicity. All analyses included 25 participants without missing data for the variables examined.

Abbreviations: ART, antiretroviral therapy; CI, confidence interval; CRP, C-reactive protein; FDR, false discovery rate; GLS, global longitudinal strain; HIV, human immunodeficiency virus; LV, left ventricular; QTc, heart rate-corrected QT interval; Tp-e, interval from the peak to the end of the T wave.

Supplementary Table S5. Descriptive comparison of cardiac measures according to virological status among people with HIV.

Variable	Newly diagnosed/viremic PWH (n = 5)	Virologically suppressed PWH (n = 20)
LV GLS, %	−17.5 [−17.8 to −15.5]	−17.1 [−17.5 to −16.0]
Tp-e interval, ms	72 [70–78]	74 [72–76]
Tp-e/QTc ratio	0.182 [0.179–0.185]	0.186 [0.180–0.189]

Data are presented as median [interquartile range]. Given the very small size of the newly diagnosed/viremic subgroup (n = 5), these comparisons were considered descriptive and exploratory, and no inferential conclusions were drawn.

Supplementary Table S6. Intraobserver and interobserver reproducibility of LV GLS and Tp-e measurements

Parameter	Reproducibility	n	Mean bias*	95% limits of agreement	ICC
Tp-e, ms	Intraobserver	20	0.20	−0.83 to 1.23	0.995
Tp-e, ms	Interobserver	20	0.10	−1.44 to 1.64	0.990
LV GLS, %	Intraobserver	20	0.07	−0.15 to 0.29	0.998
LV GLS, %	Interobserver	20	0.00	−0.24 to 0.24	0.999

Mean bias was calculated as the difference between the repeated and original measurements. Limits of agreement were calculated as mean bias ± 1.96 SD of the differences. ICC, intraclass correlation coefficient; LV GLS, left ventricular global longitudinal strain; Tp-e, T-peak to T-end interval

Supplementary Figure S1

(A) Normal P-P plot of standardized residuals for the LV GLS model

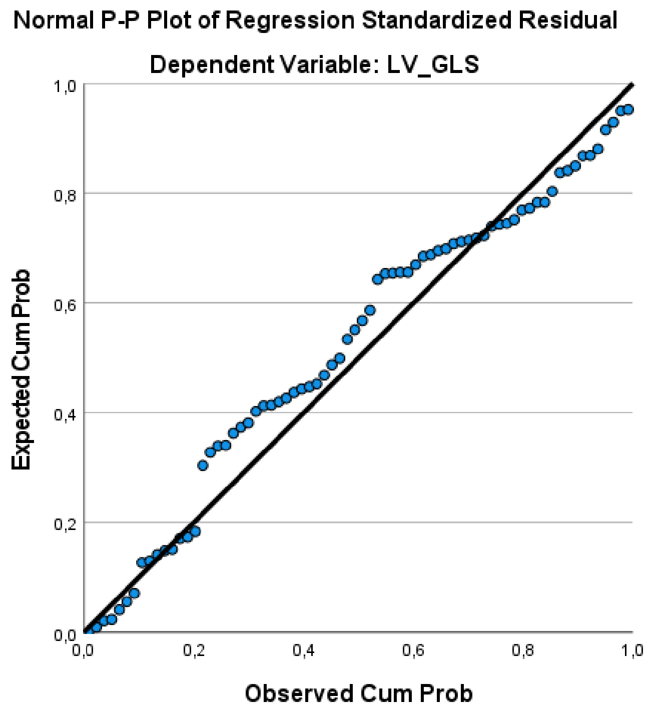

(B) Standardized residuals versus standardized predicted values for the LV GLS model

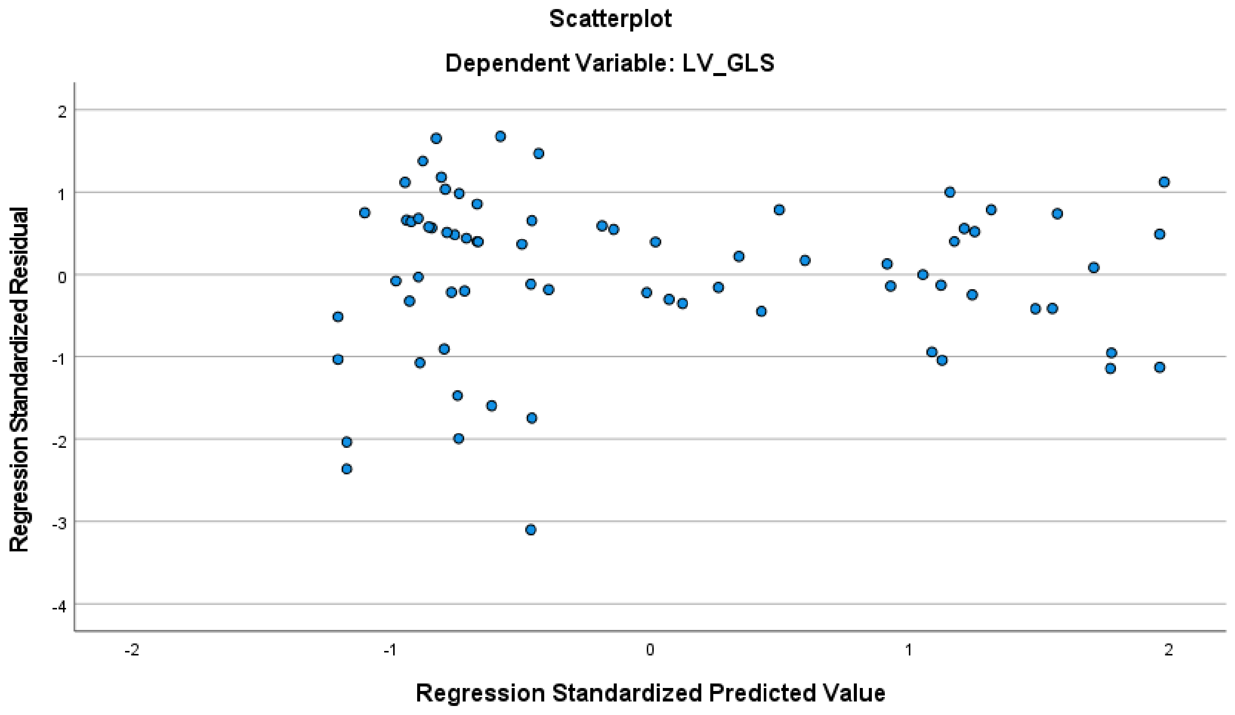

(C) Normal P-P plot of standardized residuals for the Tp-e interval model

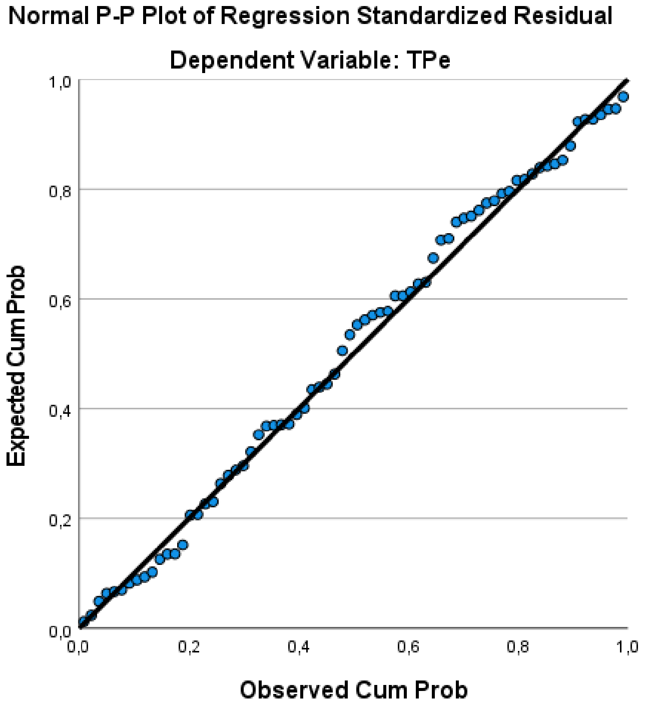

(D) Standardized residuals versus standardized predicted values for the Tp-e interval model

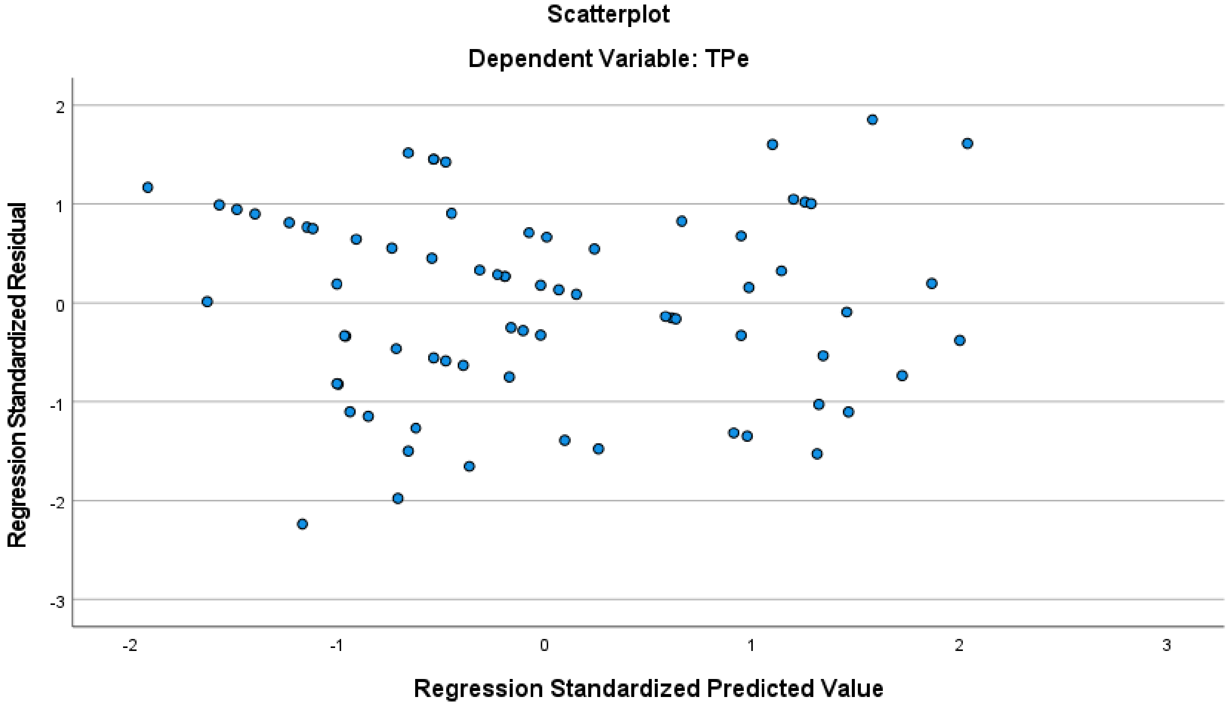

(E) Normal P-P plot of standardized residuals for the Tp-e/QTc ratio model

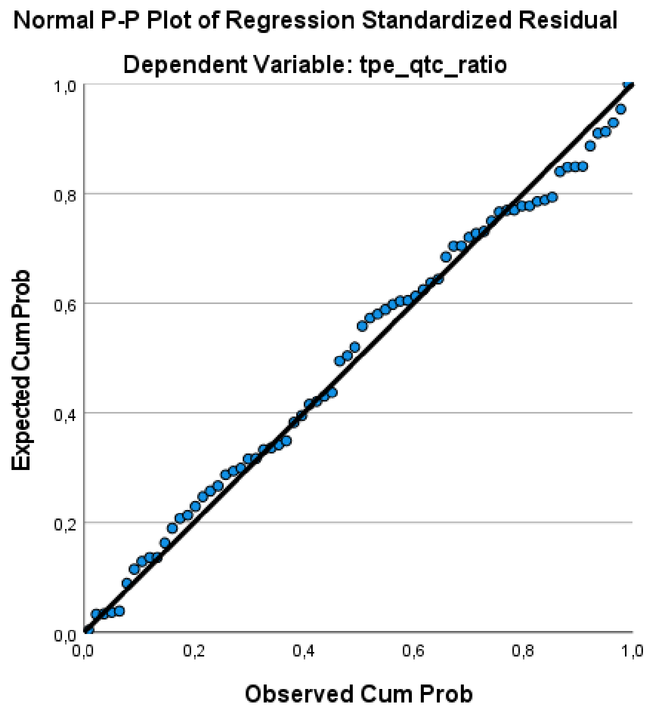

(F) Standardized residuals versus standardized predicted values for the Tp-e/QTc ratio model

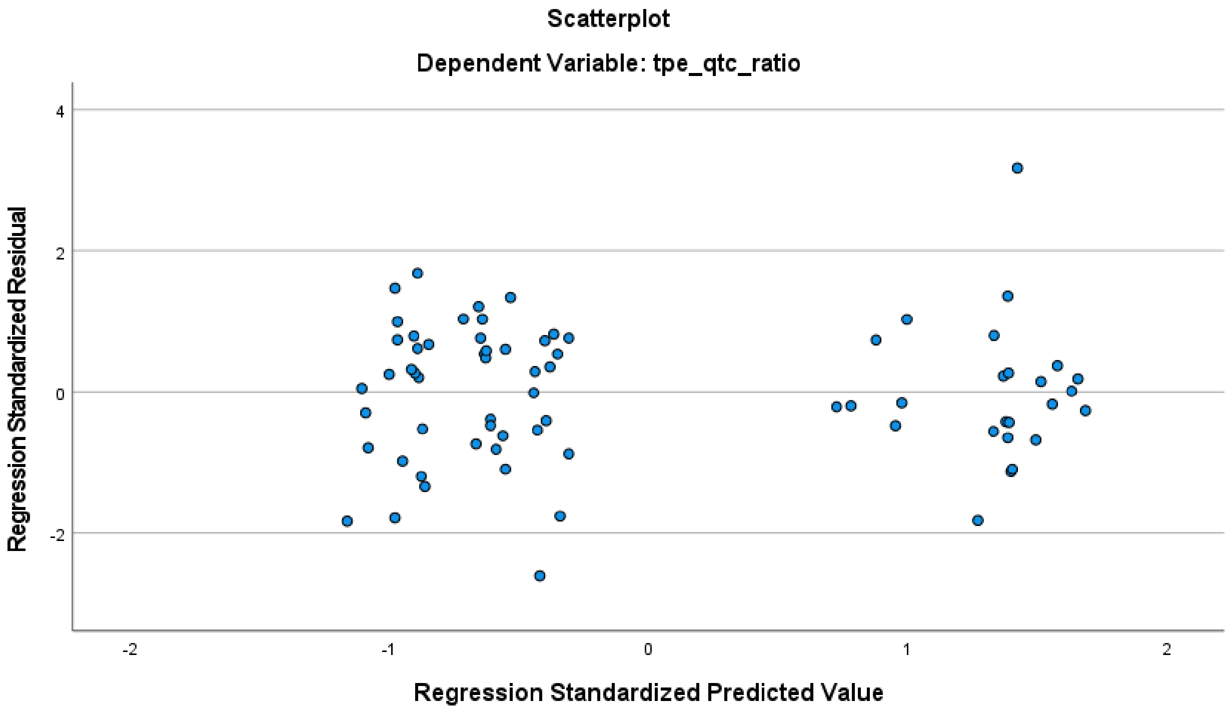

Supplementary Figure S1. Regression diagnostic plots for the principal multivariable linear regression models. Normal P-P plots of standardized residuals and plots of standardized residuals against standardized predicted values are shown for the models with LV GLS (A-B), Tp-e interval (C-D), and Tp-e/QTc ratio (E-F) as dependent variables. The models were adjusted for HIV status, age, sex, current smoking, and hyperlipidemia.
